# Hyaluronan regulates synapse formation and function in developing neural networks

**DOI:** 10.1038/s41598-020-73177-y

**Published:** 2020-10-05

**Authors:** Emily Wilson, Warren Knudson, Karen Newell-Litwa

**Affiliations:** Department of Anatomy and Cell Biology, Brody School of Medicine, East Carolina University, Greenville, NC USA

**Keywords:** Cellular neuroscience, Synaptic plasticity, Inhibition-excitation balance

## Abstract

Neurodevelopmental disorders present with synaptic alterations that disrupt the balance between excitatory and inhibitory signaling. For example, hyperexcitability of cortical neurons is associated with both epilepsy and autism spectrum disorders. However, the mechanisms that initially establish the balance between excitatory and inhibitory signaling in brain development are not well understood. Here, we sought to determine how the extracellular matrix directs synapse formation and regulates synaptic function in a model of human cortical brain development. The extracellular matrix, making up twenty percent of brain volume, is largely comprised of hyaluronan. Hyaluronan acts as both a scaffold of the extracellular matrix and a space-filling molecule. Hyaluronan is present from the onset of brain development, beginning with neural crest cell migration. Through acute perturbation of hyaluronan levels during synaptogenesis, we sought to determine how hyaluronan impacts the ratio of excitatory to inhibitory synapse formation and the resulting neural activity. We used 3-D cortical spheroids derived from human induced pluripotent stem cells to replicate this neurodevelopmental window. Our results demonstrate that hyaluronan preferentially surrounds nascent excitatory synapses. Removal of hyaluronan increases the expression of excitatory synapse markers and results in a corresponding increase in the formation of excitatory synapses, while also decreasing inhibitory synapse formation. This increased excitatory synapse formation elevates network activity, as demonstrated by microelectrode array analysis. In contrast, the addition of purified hyaluronan suppresses excitatory synapse formation. These results establish that the hyaluronan extracellular matrix surrounds developing excitatory synapses, where it critically regulates synapse formation and the resulting balance between excitatory to inhibitory signaling.

## Introduction

From the beginning of brain development, hyaluronan (HA) critically regulates neural circuit formation. At the onset of neurulation, HA is necessary for neural crest cell migration^1,2^. Later in cortical neurogenesis, HA regulates neural progenitor cell proliferation and promotes neuronal differentiation, migration and formation of cortical layers^3,4^. Once neural circuits have formed, HA surrounds neural cells and regulates their activity, most notably in peri-neuronal nets surrounding a subset of inhibitory neurons^5–7^. In spite of these critical roles for HA in neural circuit formation and function, HA has long been thought to be absent from synapses between neurons^8,9^. Fractionated synaptosomes revealed no morphologic changes in response to enzymatic digestion of HA^9^. However, these studies focused on mature synapses, thus it is still unclear whether HA regulates synapse formation early in neurodevelopment. Given the other critical roles for HA in early neurodevelopment, we sought to determine whether HA regulates the initial formation of synaptic contacts between neurons and the resulting development of spontaneous neural activity.

To better understand how HA impacts early brain development, it is important to understand how HA orchestrates extracellular matrix (ECM) organization. HA is the main component of the ECM, which accounts for 20% of the brain volume^8,10^. HA itself is a glycosaminoglycan chain consisting of repeating disaccharides measuring up to 10^6^–10^7^ Da^8^. HA is synthesized at the cellular membrane by transmembrane hyaluronan synthases (HAS)^11^. Of the three hyaluronan synthases, HAS2 and HAS3 are the most abundant in the brain^3,12^. Specifically, HAS2 produces the majority of HA within the mouse cortex, whereas HAS3 is responsible for HA production in the hippocampus^12^. HAS proteins form a pore within the plasma membrane, through which the newly synthesized HA is extruded directly into the extracellular space^13^. As the HA is secreted into the ECM it is bound by lectin domains of proteoglycans, such as neurocan, brevican, aggrecan and versican, which are also known as lecticans^8^. Neurocan and brevican are brain specific lecticans, while versican and aggrecan are ubiquitously expressed^14^. These proteoglycans consist of a protein core with attached chondroitin sulfates and binding proteins, known as HA binding link proteins (HAPLNs) that link them to the HA backbone^6,8,15^. These supramolecular HA-proteoglycan complexes are responsible for maintaining space and structure through extensive interactions with water. HA-proteoglycan complexes are capable of binding three layers of water molecules around them^16^. In addition to its spatial properties, this meshwork interacts with other ECM components, such as collagen, Tenascin C, fibronectin, and laminin^15^. HA can also regulate cell function through interaction with cell surface receptors^8^. The dominant cell surface receptor for HA is CD44^17^. CD44-HA interactions regulate cellular functions, including cell migration, proliferation and differentiation^18–24^. These combined physical and biochemical properties of HA allow it to orchestrate numerous physiological processes.

Conversely, disruption of the HA-based ECM contributes to pathological conditions. Reductions in brain HA levels in the hippocampus result in hyperexcitability and contribute to the development of epileptiform activity^12,25^. A conditional knockout mouse for Hyaluronan Synthase 2 (HAS2) significantly decreases HA levels in the cortex, resulting in epileptic seizures^12^. Similarly, HA removal via hyaluronidase injection into the right lateral ventricle of cat brains results in epileptiform activity^16^. Hyperexcitability of neural networks is characteristic of the neurodevelopmental disorders of epilepsy, intellectual disability, and autism spectrum disorders ^26–28^. In the following research, we focus on the role of HA in synapse formation and synaptic alterations underlying neurodevelopmental disorders. Through direct manipulations of HA levels, we sought to determine whether HA regulates the formation of neural networks and their activity, and whether altered HA levels contribute to disease-associated hyperexcitable states.

Neuronal activity reflects a balance between excitatory and inhibitory neurotransmission. The majority of neurotransmission occurs at specialized synaptic connections. Excitatory synapses promote action potential formation, whereas inhibitory synapses suppress action potential formation^29^. In mammals, synapse formation and spontaneous neural activity begin around mid-fetal gestation. At the onset of synaptogenesis, excitatory synapses form along the dendritic shaft and actin-rich dendritic protrusions, known as spine precursors. In post-natal brain development, these spine precursors mature into the familiar mushroom-shaped spines, which are also enriched for actin^30^. At excitatory synapses, glutamate is the predominant neurotransmitter. Vesicular Glutamate Transporter (vGlut) packages glutamate into pre-synaptic vesicles^29^. When glutamate is released into the synaptic cleft, it binds to post-synaptic glutamate receptors. These glutamate receptors are clustered into an electron-dense scaffold, known as the post-synaptic density (PSD)^31^. Scaffolding proteins, such as Post-Synaptic Density-95 (PSD-95), connect glutamate receptors with the machinery necessary for action potential formation^32^. In contrast to excitatory synapses, inhibitory synapses form along the dendritic shaft, somata, and axonal initial segments, and this localization persists in post-natal development^33^. In addition to the absence of post-synaptic dendritic projections, inhibitory synapses also lack post-synaptic actin-enrichment and the electron-dense PSD^34^. The predominant inhibitory neurotransmitter is GABA^35^. Vesicular GABA Transporter (VGAT) packages GABA into synaptic vesicles at inhibitory nerve terminals^36^. At the post-synaptic side of inhibitory synapses, gephyrin scaffolds GABA receptors and downstream signaling components^29^. The balance between excitatory and inhibitory neurotransmission is crucial for cognitive functions, as is clearly demonstrated by alterations in the ratio between excitatory and inhibitory (E/I ratio) in brain disorders. Intriguingly, the majority of brain disorders exhibit hyperexcitability^26,27,29^, resulting in epileptic seizures, learning disabilities, and neuronal cell loss leading to neurodegeneration^16,26,27,29,37,38^.

Breakthroughs in understanding the emergence of neuronal disorders have been achieved utilizing 3-dimensional (3-D) human brain models, such as organoids and spheroids^39^. Cortical spheroids differ from whole brain cerebral organoids as they are brain-region specific^40^. By three months in culture, pre-frontal cortical spheroids exhibit characteristic ventricles, or zones of proliferation, which house the neural progenitor cell pool^39^. Radial glia project from these regions, allowing for the migration of developing neurons to the cortical plate, a region corresponding to the cortical layers^40^. These human models offer an unprecedented research model to identify molecular mechanisms that regulate the development of neuronal activity and alterations that result in disease-associated hyperexcitability signatures^39^. Here we establish a human brain spheroid model of the HA-based ECM and use this model to investigate how HA alterations disrupt neural circuitry at the level of synapse formation and function. The following research tests whether the hyaluronan-based extracellular matrix directs excitatory synapse formation and regulates the resulting neuronal activity.

In later development, HA surrounds and stabilizes mature synapses^8^. However, whether HA orchestrates the initial formation of synapses is unknown. Our data from the onset of the synaptogenesis demonstrates that HA is present and enriched at nascent excitatory synapses as compared to inhibitory synapses. At these developing excitatory synapses, HA antagonizes excitatory synapse formation and suppresses the corresponding neural activity. These changes at excitatory synapses inversely correlated with inhibitory synapse formation. These findings establish HA as a crucial regulator of synapse formation and function.

## Materials and methods

### Cell lines

Neurotypic control skin fibroblasts of the cell line 7545 19B (cortical spheroids) were reprogrammed into human-induced pluripotent stem cells (hIPSCs) in the laboratory of Dr. Mike McConnell (UVa) with the addition of Yamanaka transcription factors Oct3/4, Sox2, Klf4, and c-Myc using the CytoTune-iPS 2.0 Sendai Reprogramming Kit (Invitrogen). 7545 fibroblasts were obtained under an MTA with the Coriell Institute. 9319 and BOH1 hIPSCs (for 2D differentiated cultures) were obtained under a material transfer agreement (MTA) with Kristen Brennand and the Salk Institute.

hIPSCs were maintained in Essential 8 Medium + E8 supplement (Gibco) on hESC Matrigel (Corning) coated plates. Upon splitting, 10 μM of the ROCK inhibitor, Y27632 (Selleck Chemicals), was added to the cell medium.

### 3D cortical spheroid culture

Cortical spheroids were produced following an adaption to Pasca methods, Nature Methods 2015^41^. Briefly, enzymatically lifted hIPSCs were transferred to ultra-low attachment plates and cultured in DMEM supplemented with Knockout Serum Replacement (Gibco) supplemented with 5 μM Dorsomorphin (BioVision), 10 μM SB431542 (Miltenyi Biotec), 10 μM Y27632 (Selleck Chemicals) for 6 days. The resulting spheroids were then maintained in neurobasal media until day 90: Neurobasal A medium, 2% B-27 supplement without vitamin A, GlutaMAX L-glutamine supplement (Gibco) penicillin/ streptomycin (Gibco). Spheroids were next supplemented with 20 ng/mL of bFGF and EGF (PeproTech) from day 6 to 25, and 20 ng/mL of BDNF and NT3 (Shenandoah Biotechnology) from day 26 to 42. Spheroids were harvested beginning at day 90 for analysis (refer to Fig. 3 for experimental details).

### 2D differentiated monolayer culture

Neural Progenitor Cells (NPC) were generated from embryoid bodies according to Brennand et al. 2011. NPCs were differentiated or maintained for one week before analysis. NPCs were plated at 50,000 cells per well onto 12 well plates (Corning), precoated with polyornithine (Sigma Aldrich) and laminin (Corning), in NPC media: DMEM/F-12 + GlutaMAX, 1% N-2 Supplement, and 2% B27 without vitamin A (Gibco), 1 μg/mL laminin (Corning) and 20 ng/mL basic fibroblast growth factor (bFGF) (PeproTech). After 24 h NPCs were either differentiated toward neurons (neural differentiation media) or astrocytes (astrocyte differentiation media), or maintained in NPC media. Media was changed every other day for one week before analysis.

Neural differentiation media: DMEM/F-12 + GlutaMAX, 1% N-2, 2% B-27 with vitamin A (Gibco), 20 ng/mL BDNF, 20 ng/mL GDNF (Shenandoah Biotechnology Inc), 400 μM cAMP (Sigma Aldrich), and 200 nM ascorbic acid (Fisher Scientific).

Astrocyte differentiation media: Astrocyte Medium (Cat No. 1801, ScienCell) supplemented with penicillin/streptomycin, FBS and astrocyte growth supplement (AGS).

### ECM manipulation

High Molecular Weight Sodium Hyaluronate, 1.0–1.8 MDa, (HA 15 M-5 LifeCore) was dissolved in neurobasal medium for 24 h before use. Hyaluronan was added to culture medium at 250ug/mL. Streptomyces Hyaluronidase (Sigma Aldrich 37259-53-3) was added directly to culture medium at 10 U/mL. Treatment lasted 24 h before harvesting for analysis or fixing for immunohistochemistry. Adenoviral constructs were used for the overexpression of HAS2 in cortical spheroids (Fig. S3). The constructs used were prepared in the same manner as Ishizuka et al.^42^.

### Immunohistochemistry

Cortical spheroids were fixed in 4% paraformaldehyde for 24 h and placed in 30% sucrose for 24 h. Spheroids were then embedded in OCT mounting media overnight (Sakura Finetek USA), flash frozen, and cryosectioned into 10 μm thick sections. Cryosections were permeabilized with 0.2% TritonX-100 in 1 × PBS before immunostaining. Primary antibodies were diluted in 2% normal goat serum in PBS, added to fixed cultures and kept at 4 °C overnight. After three PBS washes secondary antibodies diluted in 2% normal goat serum in PBS were added to fixed cultures and kept at room temperature for 1 h. Cryosections were mounted using Fluoro-gel II with DAPI mounting medium (Electron Microscopy Sciences) for confocal imaging or Vectashield without DAPI (Vector Laboratories 101098-042) for STORM imaging.

2D cultures were fixed after 1 week in 4% paraformaldehyde, 4% sucrose, 1 × PBS. Cells were permeabilized with 0.2% TritonX-100 in 1 × PBS before staining. Primary antibodies were diluted in 2% normal goat serum in PBS, added to fixed cultures and kept at 4 °C overnight. After three PBS washes secondary antibodies diluted in 2% normal goat serum in PBS were added to fixed cultures and kept at room temperature for 1 h. Cultures were mounted using Fluoro-gel II with DAPI mounting medium (Electron Microscopy Sciences).

HA was visualized using Hyaluronic Acid Binding Protein (HABP). HABP is a biotinylated link protein G1 domain of the proteoglycan versican, which binds selectively to HA. Fluorescently labeled streptavidin was used to identify HABP. See supplemental tables 1 and 2 (in the Supplementary tables) for further information. After immunostaining, cryosections were subsequently imaged using a ZEISS LSM 700 confocal or Nikon Ti2-E inverted STORM microscope. All immunohistochemistry was performed in triplicate on at least three different sets of spheroids.

### Confocal microcopy

Cortical spheroid sections were imaged on a Zeiss LSM 700 confocal microscope at 40 × total magnification, using the 639, 555, 488 and 405 channels. Z-stacked images were acquired, 5 images across 5 μm, and merged in ImageJ to generate maximum intensity projections. Confocal images were further analyzed using ImageJ 1.52a analysis software (https://imagej.nih.gov/ij/). Each 4-channel image was analyzed as an 8-bit tiff-file. All samples of the same experiment had equal threshold values for each channel. 10 area samples measuring 100 μm in diameter were used to determine the intensity and density of the fluorescent markers along the edge of the cortical spheroid, coinciding with the cortical plate location. Spheroids were stained with pre- and post-synaptic markers. To analyze the total synapse area, we used the ImageJ colocalization plug in. This produced an image indicating areas where thresholded pre- and post-synaptic markers colocalized by intensity ratio of 10% or more. The area of each individual marker as well as the colocalized synapse area was measured and normalized to DAPI. Normalization to nuclei density (DAPI) has been performed in other brain organoid studies to compare regions of similar cell densities^43,44^, and to evaluate synaptic changes^45^.

### STORM microscopy

STochastic Optical Reconstruction Microscopy (STORM) Images of cortical spheroids were acquired with a Nikon Ti2-E inverted microscope with an L-APPS H-TIRF attachment and 4-line (405 nm, 488 nm, 561 nm, 640 nm) LUN-F laser module and 100X 1.49NA Apo TIRF objective. The raw images are collected with the 3D STORM lens to a back-thinned Princeton Instruments Pro-EM-HS EMCCD 512 × 512 camera and acquired and analyzed with NIS-Elements STORM modules. Processed .nd2 files were analyzed in ImageJ. .nd2 files were opened as a hyperstack, 3D projection was then used to plot the brightest point with the Y-axis as the axis of rotation. A Gaussian blur filter with a radius of 2.00 pixels was applied to avoid bias. A 10 pixel line width was used to analyze of the intensity of synaptic molecules using the plot profile function of ImageJ. Please note that the intensity profile in Fig. 2 reflects the number of molecular events detected by STORM analysis, rather than arbitrary fluorescence units^46^.

### Microelectrode array analysis

For MEA analysis, spheroids at day 76 were dissociated using a Primary Neuron Isolation Kit (Thermo Scientific Pierce MAN0016221) and plated onto PEI-coated MEA plates prepared as described below. An Axion BioSystems Maestro Edge Multielectrode Array System (MEA) was used to record spontaneous action potentials in dissociated spheroids. MEA plates were prepared 48 h before cells were plated as follows: Wells were incubated 1 h at 37 °C with 0.1% polyethylenimine in ddH_2_O and rinsed 4 times with sterile water and left at room temperature overnight. After 24 h, 5 μg/mL laminin was added to wells and left overnight at room temperature. Wells were washed twice with sterile PBS before 250,000 cells/well of dissociated spheroids were added. Cultures were given MEA medium consisting of Neurobasal A medium, 2% B-27 Plus supplement without vitamin A, glutaMAX l-glutamine supplement (Gibco) penicillin/ streptomycin (Gibco). Cells were acclimated to the plates for 2 weeks before treatment and recording. Manipulation of HA levels was conducted for 24 h. Wells were recorded 10 min/h for 24 h. After treatments wells were given fresh media. Washout was recorded for 24 h. (10 min/h). Wells were treated with 4 μM tetrodotoxin (TTX) and immediately recorded for 10 min to suppress action potential formation and verify that the recorded electrical currents correspond to spontaneous neural activity. MEA experiments were performed on 6 sets of dissociated spheroids.

### Statistical analyses

Statistical analyses were performed using SigmaPlot 13.0 Software. Data sets were first tested for normality using Shapiro–Wilk tests. T-tests were run on parametric data to determine statistical significance. Non-parametric data was analyzed using Mann–Whitney Rank Sum tests to test for statistical significance.

### mRNA nanostring analysis

Day 91 organoids (after 24 h of acute HA treatment) were harvested for RNA analysis. RNA was obtained using a Nucleospin RNA/Protein Isolation kit (Macherey–Nagel 740933.50). RNA concentration was quantified using a NanoDrop One spectrophotometer. 50–100 ng of total RNA was hybridized with reporter and capture probes for nCounter Gene Expression code sets (Neuropathology Codeset) according to the manufacturer’s instructions (NanoString Technologies). Using the nSolver analysis system data were normalized to spiked positive controls and housekeeping genes. Transcript counts less than the mean of the negative control transcripts plus 2std for each sample were considered background. Nanostring mRNA analysis was performed on RNA harvested from three sets of cortical spheroids.

## Results

### Characterization and manipulation of hyaluronan in a human cortical spheroid model

**Figure 1:**
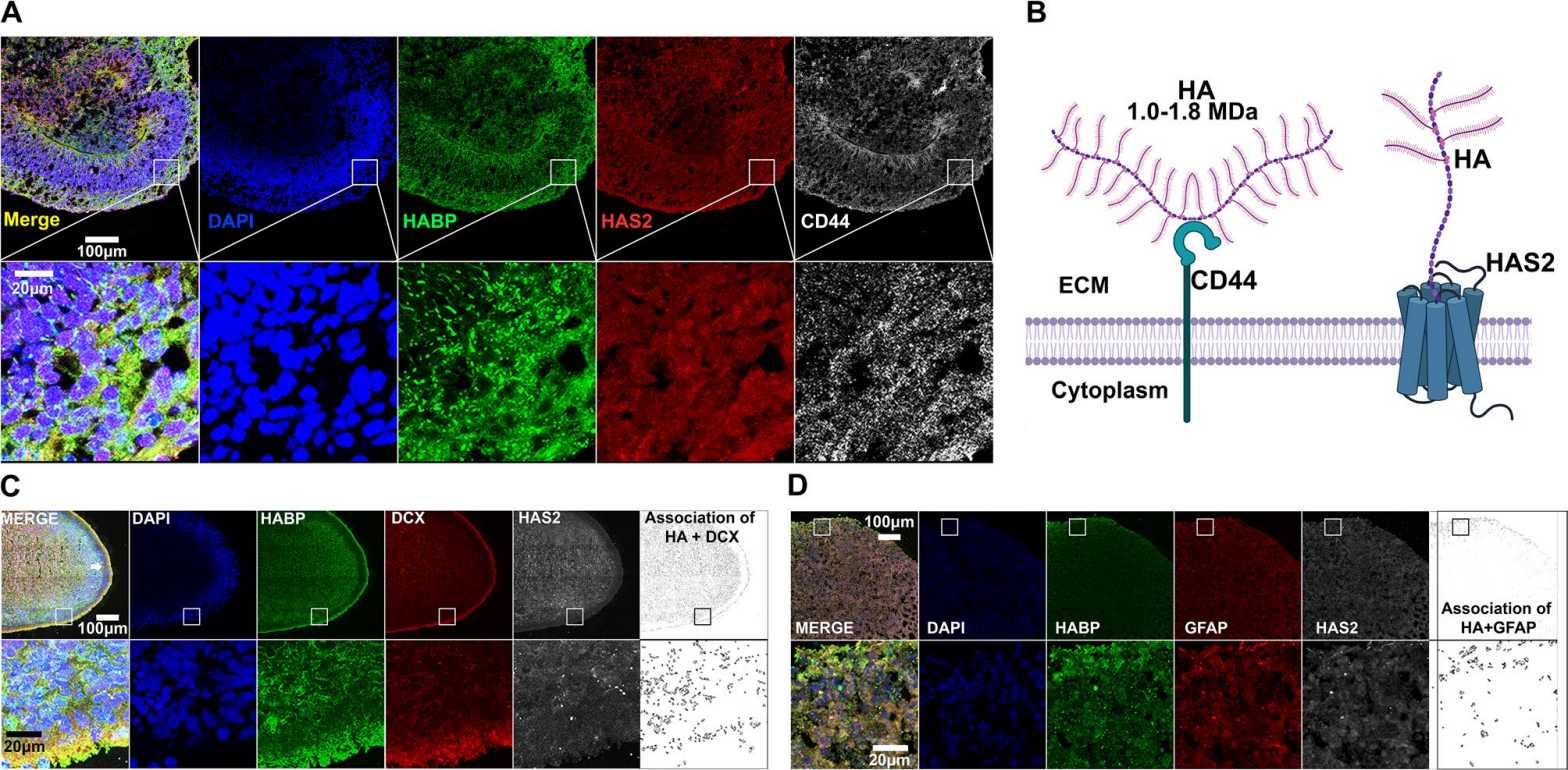
Human IPSC-derived Cortical Spheroids Produce Hyaluronan ECM. (**A**) 10 μm thick cryosections of 90-day-old control spheroids were stained for ECM components. Left to right: merged channels, nuclei marker DAPI (blue), HA as detected by HABP (green), HA synthase HAS2 (red), and HA receptor CD44 (white). Bottom panel highlights a section of the cortical plate at increased magnification. Scale bar of top panel: 100 μm, bottom panel: 20 μm. (**B**) Graphic illustration of how HA is produced by HAS and interacts with CD44 at the cell membrane. (**C**) 10 μm thick cryosections of 90-day-old control spheroids were stained for DAPI (blue), HABP (green), neuronal marker DCX (red) and HAS2 (white). Arrows in top left panel highlight the cortical plate. Bottom panel highlights a section of the cortical plate at increased magnification. Scale bar of top panel: 100 μm, bottom panel: 20 μm. (**D**) 10 μm thick cryosections of 90-day-old control spheroids were stained for DAPI (blue), HABP (green), astrocyte marker GFAP (red) and HAS2 (white). Bottom panel highlights a section of the cortical plate at increased magnification. Scale bar of top panel: 100 μm, bottom panel: 20 μm. Further analysis of cell-type specific expression of HA-ECM components can be found in Fig. S2, while validation of HAS2 immunostaining can be found in Fig. S3.

We first established that human cortical spheroids contain the proper machinery for HA synthesis and signaling, and that they do, in fact, synthesize an HA-based ECM at the time of synaptogenesis. Consistent with others, we have previously established that discrete synapses are present after 3 months of brain spheroid culture^39^. In order to detect the presence of HA machinery, we immunostained 3-month-old cortical spheroid cryosections for the predominant HAS isoform of the cortex, HAS2, as well as the primary HA receptor, CD44. As shown in Fig. 1A, cortical spheroids endogenously express HA (detected using HABP), HA synthase, HAS2, and HA receptor, CD44 (Fig. 1A,B). Furthermore, HA machinery is enriched within the cortical plate surrounding the ventricles. As demonstrated in Fig. 1C, this region is enriched for neurons and serves as the site of synaptogenesis. In this region, HA is associated with both neurons and astrocytes (Fig. 1C,D, Fig. S2).

### Hyaluronan is present at nascent excitatory synapses

**Figure 2:**
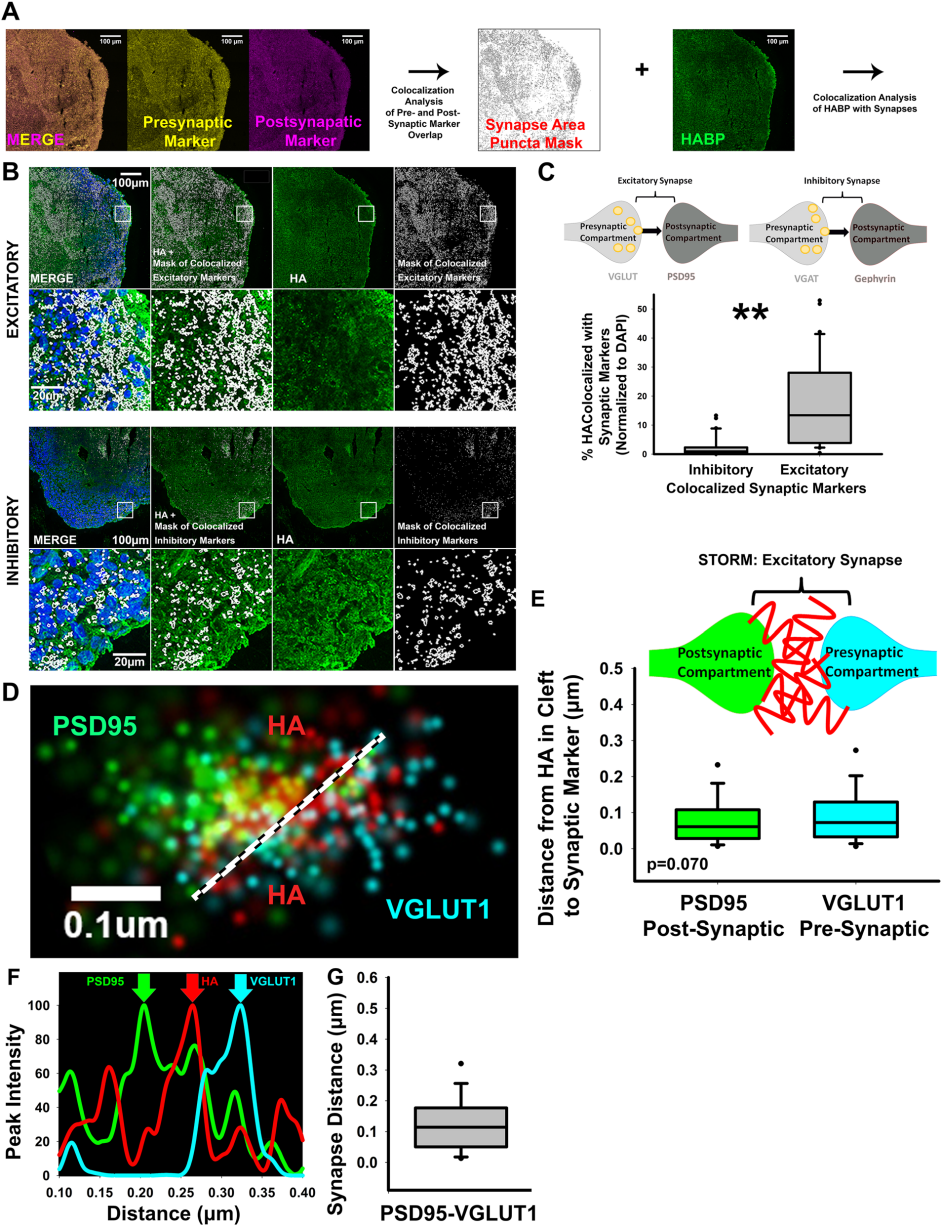
HA is present at excitatory synapses. (**A**) Representative workflow of analysis of HA localization at excitatory and inhibitory synapses. Confocal images of pre- and post-synaptic markers are first analyzed to identify co-localization in synapses. The identified synapses are then analyzed for colocalization with HA. (**B**) Representative images of DAPI (blue), and HA (green) together with excitatory synapse markers, vGlut-1 and PSD-95, and inhibitory synapse markers, vGAT and gephyrin. White outlines indicate the identified synapses. (**C**) Co-localization analysis reveals that HA is preferentially enriched at excitatory synapses, shown as %HABP colocalized with synaptic markers. The corresponding percentage of excitatory and inhibitory synapses containing HA is quantified in Fig. S4, further demonstrating that excitatory synapses preferentially associate with HA. (**D**) STORM imaging was used to visualize HA at individual excitatory synapses. The pre-synaptic marker vGlut-1 is shown in blue, HA is shown in red, and post-synaptic PSD-95 is shown in green. Note that HA is positioned between the pre- and post-synaptic compartment of the excitatory synapse. (**E**) Quantification of the distance between HA and pre-synaptic vGlut-1 and post-synaptic PSD-95 as determined by the displacement of maximum peak intensities. There is not a significant difference between the distance of HA-vGlut1 and HA-PSD-95, indicating that HA does not preferentially localize to one side of the excitatory synapse, and instead lies in the synaptic cleft between pre- and post-synaptic compartments as illustrated by the corresponding diagram. n = 167 synapses. (**F**) Representative plot profile of the excitatory synaptic markers and HA in the highlighted synapse (**D**). (**G**) Quantification of distance from pre-synaptic vGlut-1 to post-synaptic PSD-95, n = 167 synapses.

After establishing that cortical spheroids express an HA-based matrix, we next sought to determine whether HA is present at developing synapses (Fig. 2A). Using confocal imaging, we observed that HA is preferentially enriched at excitatory synapses (Fig. 2B,C, 4). To further establish where HA is present at nascent excitatory synapses, we used super-resolution STochastic Optical Reconstruction Microscopy to resolve individual excitatory synapses. The pre- and post- synaptic compartments are separated by a synaptic cleft that is approximately 20 nm wide, as measured by electron microscopy^9^. Since confocal microscopy is limited to a resolution of ~ 200 nm, it is unable to distinguish between pre- and post-synaptic compartments^47^. By contrast, STORM offers an approximately tenfold increase in image resolution, with a resolution limit of ~ 20 nm^46^. Thus, STORM microscopy enables detection of distinct pre- and post-synaptic compartments. Using STORM, we identified pre-synaptic compartments by the presence of the vesicular glutamate transporter-1 (vGlut1), the predominant glutamate transporter in our HCSs. To identify adjacent post-synaptic compartments, we immunostained for the scaffolding protein, Post-Synaptic Density-95 (PSD-95). After fluorescent labeling of excitatory synapses and HA, we used three-color STORM, which revealed that HA is present at nascent excitatory synapses. In the majority of cases, HA is sandwiched in the synaptic cleft directly between the pre- and post-synaptic compartments (Fig. 2D–F). Measurement of the peak intensity for the presynaptic vGlut-1, HA, and post-synaptic PSD-95 puncta across multiple (167) synapses demonstrated that HA is positioned equidistant from the pre- and post-synaptic compartments (Fig. 2E). Distance from the pre- to post-synaptic marker (Fig. 2G) is about 100 nm, consistent with our previous observations^48^. In contrast to studies of mature mammalian synapses, in which HA surrounds the synapse but is absent from the synaptic cleft^9,49^, this result suggests that during synapse formation, HA is uniquely positioned between pre- and post-synaptic compartments. Given this close association with pre- and post-synaptic compartments of excitatory synapses, the following experiments address whether HA regulates synaptogenesis and the corresponding emergence of spontaneous synaptic activity.

### Hyaluronan manipulations alter mRNA expression of synapse markers

**Figure 3:**
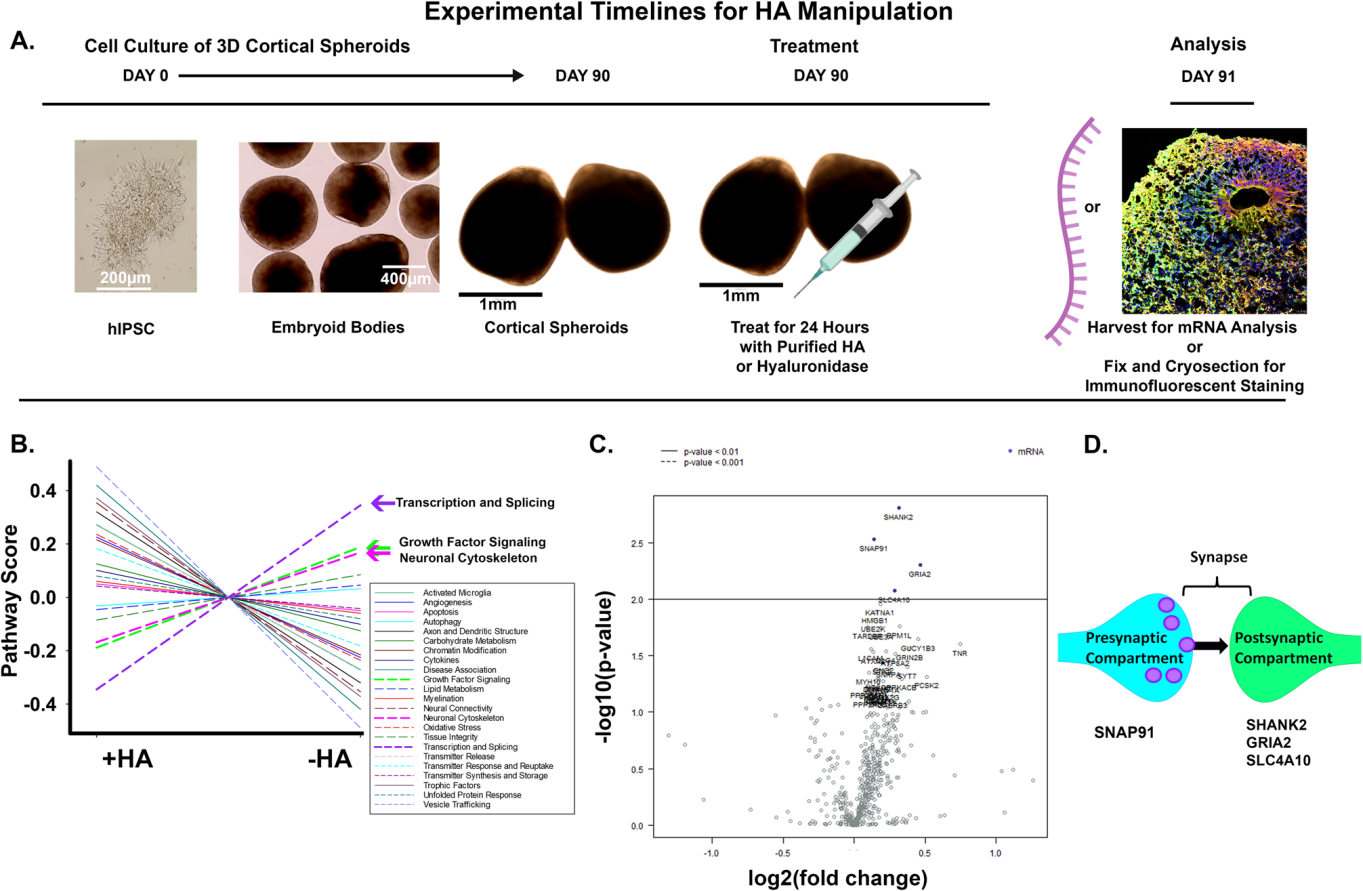
Nanostring mRNA analysis reveals synaptic changes in response to hyaluronan manipulations. (**A**) Experimental timeline for HA manipulation. Human IPSCs (Day 0) are used to make cortical spheroids (Day 90). Middle image shows intermediate stage embryoid bodies around day 9. 90-day-old spheroids are treated with purified HA or streptomyces hyaluronidase to digest HA. After 24 h of treatment, spheroids are harvested for subsequent analysis. (**B**) Average pathway scores were plotted for + HA (hyaluronan addition) and − HA (hyaluronidase treatment), revealing three pathways that were preferentially increased by hyaluronidase treatment: Transcription and Splicing (purple), Growth Factor Signaling (green), and Neuronal Cytoskeleton (pink). (**C**) Volcano plot of the differential expression of all genes analyzed. − HA differential expression is plotted against + HA differential expression, such that mRNAs to the right of 0.0 on the x-axis are upregulated in response to hyaluronidase treatment. The top four differentially-expressed mRNAs (as indicated by blue dots above the p < 0.01 line) are all synapse-associated proteins. (**D**) Representative image of a synapse showing the pre- or post-synaptic protein localization corresponding to the differentially-expressed mRNAs.

After establishing that cortical spheroids express an HA-based matrix, we sought to acutely perturb HA levels during synapse formation. In order to manipulate endogenous HA levels, we used the following experimental protocols as detailed in Fig. 3A. Day 90 spheroids reflect fetal neurodevelopment of the dorsal forebrain around 18 weeks post-conception, corresponding with the onset on synaptogenesis^39^. In order to acutely increase HA levels, day 90 spheroids were treated with purified HA. Conversely, in order to decrease HA levels, day 90 spheroids were treated with streptomyces hyaluronidase to digest HA. After 24 h of treatment, spheroids were processed for subsequent analysis. We used HABP staining to confirm that we had successfully manipulated HA levels (Fig. S1). Notably, hyaluronidase significantly decreased HA levels, whereas purified HA incorporated into the spheroids and significantly increased HA levels (Fig. S1), thus demonstrating that we are able to acutely manipulate the HA levels of our system.

To initially examine the neural pathways impacted by acute hyaluronan perturbations in an unbiased fashion, we employed Nanostring nCounter platform to analyze transcript expression changes in over 700 mRNA targets associated with neuropathologies. mRNA was harvested from 90-day-old cortical spheroids and was subjected to Nanostring nCounter mRNA analysis using the Neuropathology panel. Unlike other targeted transcript analyses, Nanostring directly measures mRNA levels, and does not require intermediate cDNA synthesis. This increases the sensitivity of the Nanostring nCounter platform for low-abundance transcripts, while also preventing the introduction of artifacts through cDNA synthesis^50^. We compared pathway scores between groups of genes known to be involved in neuropathology (Fig. 3B). Pathway scores were plotted for + HA (hyaluronan addition) and − HA (hyaluronidase treatment), revealing three pathways that were preferentially increased in response to hyaluronidase treatment: Transcription and Splicing, Growth Factor Signaling, and Neuronal Cytoskeleton (Fig. 3B). This suggests that HA manipulation is sufficient to elicit changes in neuronal physiology. We then compared individual transcript expression levels to further determine the effects of HA manipulation. To visualize the differential expression of individual mRNA transcripts, we plotted transcript level fold change between hyaluronan removal versus hyaluronan addition in a volcano plot (Fig. 3C). Individual transcripts with significantly altered expression in response to hyaluronan manipulation are all associated with synapses, including *SNAP91*, *SLC4A10*, *SHANK2*, and *GRIA2*, all four of which are upregulated in response to HA degradation (Fig. 3D). At the pre-synaptic compartment, SNAP91 is involved in clathrin coat assembly, neurotransmitter secretion, and synaptic vesicle budding from the pre-synaptic endocytic zone membrane^51^. Within the excitatory post-synaptic compartment, SHANK2 is a post-synaptic scaffolding protein and GRIA2 is glutamate ionotropic receptor AMPA type subunit, both of which mediate excitatory neurotransmission^52,53^. Also in the post-synaptic compartment, SLC4A10 is associated with sodium channels in principal and inhibitory neurons^54^. These data demonstrate that acute HA manipulation for 24h has immediate and detectable effects on synapse-associated transcripts. The following aims address whether these synaptic mRNA transcript changes correspond with altered synapse formation and function.

### Acute HA manipulation alters excitatory synapse formation

**Figure 4:**
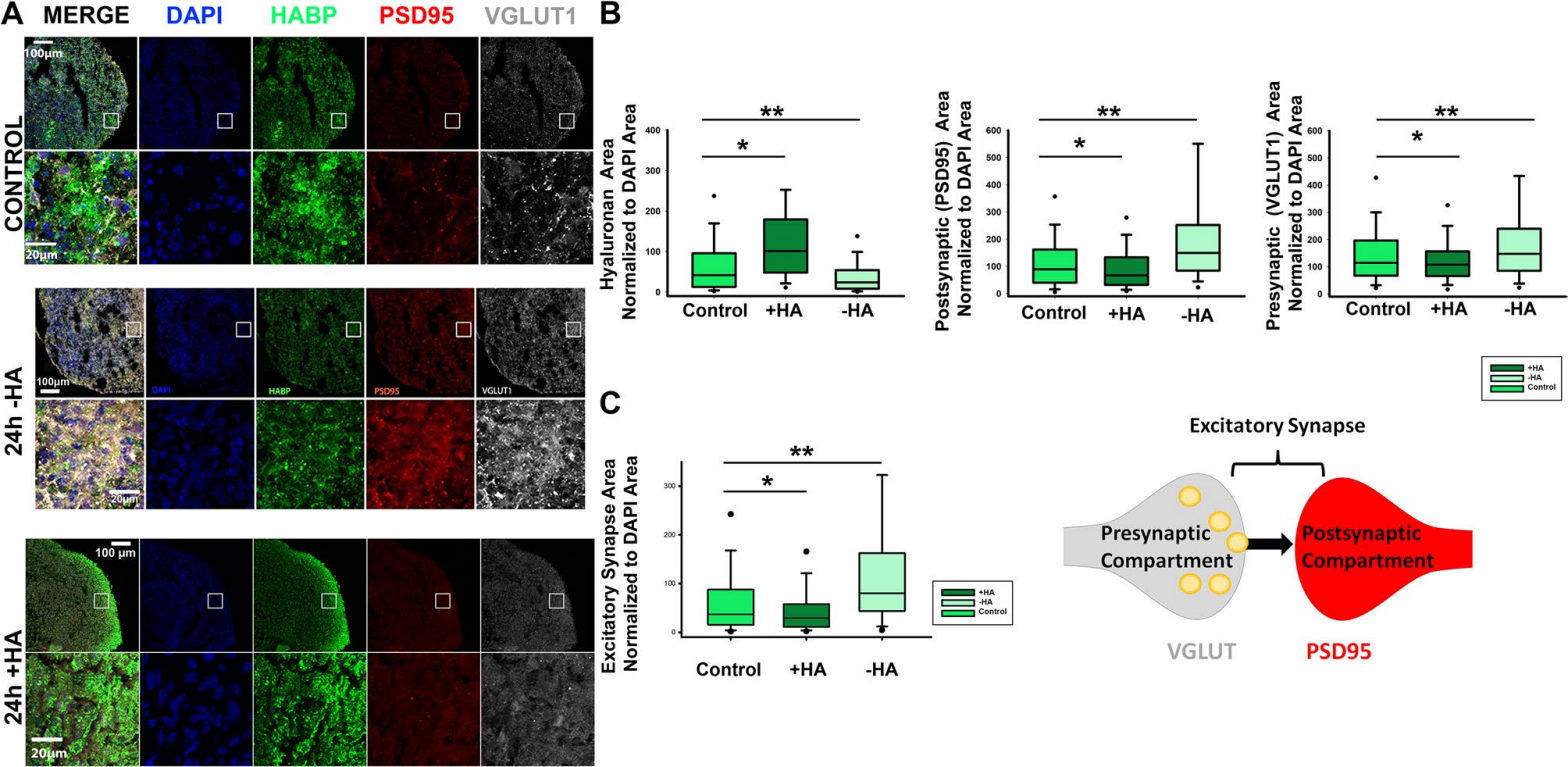
HA regulates excitatory synapse formation. (**A**) 10 μm-thick cryosections of 90-day-old cortical spheroids were stained for excitatory synapse markers after 24 h of treatment with purified HA (+ HA) or streptomyces hyaluronidase (− HA). Blue: DAPI, Green: HABP (HA), Red: PSD-95 (post-synaptic marker), Grey: vGlut-1 (pre-synaptic marker). Scale bars for top panels of spheroid images are 100 µm, scale bars for bottom ROI panels for spheroid images are 20 µm. (**B**) Graphs quantifying the area of individual synapse markers following hyaluronan manipulations. n = 30 spheroid slices per treatment. (**C**) Quantification of the resulting changes in total excitatory synapse area as determined by co-localization of pre- and post-synaptic markers normalized to DAPI. n = 30 spheroid slices per treatment. Solid black dots represent 95th percentile outliers, one asterisk signifies p value < 0.05, two asterisks signify p value < 0.01.

To assess whether HA manipulations alter the formation of excitatory synapses, we similarly treated 90-day-old cortical spheroids with either hyaluronidase to remove hyaluronan or the addition of purified high molecular-weight HA (1.0–1.8 MDa) for 24 h (Fig. 3A). This high molecular weight HA is consistent with endogenous neural HA, which is 1–10 MDa^8^. As noted above, this acute 24h treatment was sufficient to induce significant changes in the HA levels of cortical spheroids, decreasing HA levels in response to hyaluronidase, and increasing HA incorporation into extracellular matrix with the addition of purified HA (Fig. 4A,B, S1). Using immunohistochemistry, we identified excitatory synapses by the co-localization of pre-synaptic vGlut-1 and post-synaptic PSD-95 (Fig. 4). In response to decreased HA, the density of excitatory synapses, as identified by the co-localization of vGlut-1 and PSD-95, increased (Fig. 4A,C). Individually, both vGlut-1 and PSD-95 area increased in response to HA removal, demonstrating that the effects of HA removal are not specific for distinct pre- or post-synaptic compartments (Fig. 4A,B). By contrast, elevating HA levels decreased the area of vGlut-1, PSD-95, and excitatory synapses, which were identified by the co-localization of vGlut-1 and PSD-95 (Fig. 4). These results indicate that HA normally functions to restrict excitatory synapse formation.

### Acute HA manipulations result in corresponding changes in inhibitory synapse formation

**Figure 5:**
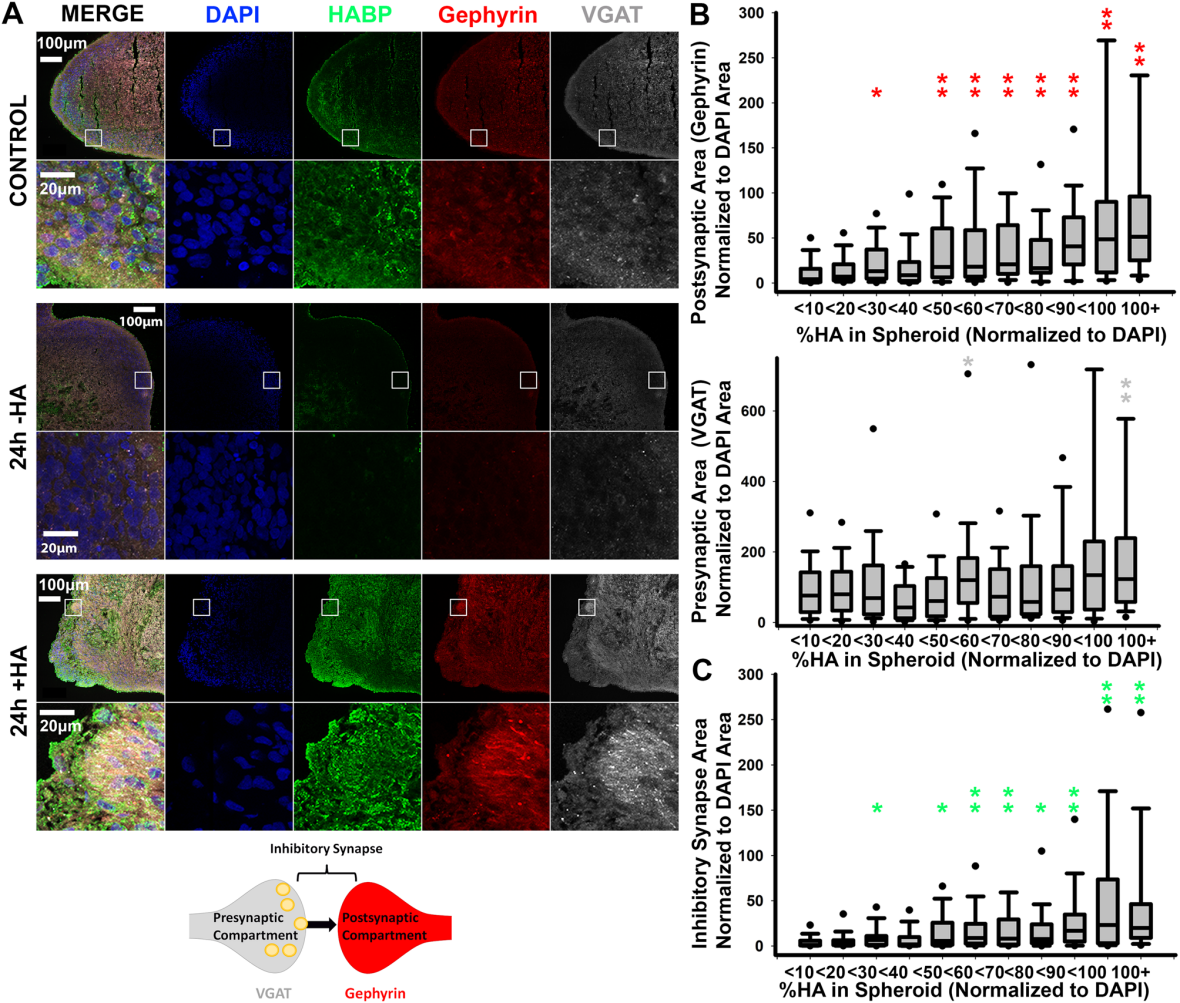
HA regulates inhibitory synapse formation. (**A**) 10 μm-thick cryosections of 90-day-old cortical spheroids were stained for inhibitory synapse markers after 24 h of treatment with purified HA (+ HA) or streptomyces hyaluronidase (− HA). Blue: DAPI, Green: HABP (HA), Red: Gephyrin (post-synaptic marker), Grey: vGAT (pre-synaptic marker). (**B**) HA levels were quantified across all treatment groups, and were used to bin the corresponding areas of inhibitory synapse marker, gephyrin and vGAT. (**C**) Quantification of the resulting changes total inhibitory synapse area as determined by co-localized pre- and post-synaptic markers normalized to DAPI. Inhibitory synapse area is stratified based on the corresponding HA levels. n = 30 spheroid slices per treatment. Solid black dots represent 95^th^ percentile outliers, one asterisk signifies p value < 0.05, two asterisks signify p value < 0.01.

To determine whether these effects on excitatory synaptogenesis lead to corresponding changes in inhibitory synapse formation, we immunostained cortical spheroid cryosections for inhibitory synapse markers. We used the vesicular GABA transporter (vGAT) to identify the pre-synaptic terminal of inhibitory synapses, and the scaffolding protein, gephyrin, to identify post-synaptic compartments of inhibitory synapses (Fig. 5A). In contrast to the effects on excitatory synapse formation in response to hyaluronan manipulations (Fig. 4), we observed opposite effects on inhibitory synapse formation (Fig. 5). We stratified all cortical spheroids based on HA levels. This analysis highlighted how inhibitory synapse formation directly scales with HA levels (Fig. 5C). Furthermore, at the level of individual pre- and post-synaptic markers, we observed increases in both pre-synaptic vGAT area and to a greater extent, post-synaptic gephyrin area (Fig. 5B). Thus, HA-mediated suppression of excitatory synapse formation inversely correlates with increased inhibitory synapse formation.

### Hyaluronan critically regulates neuronal excitability

**Figure 6:**
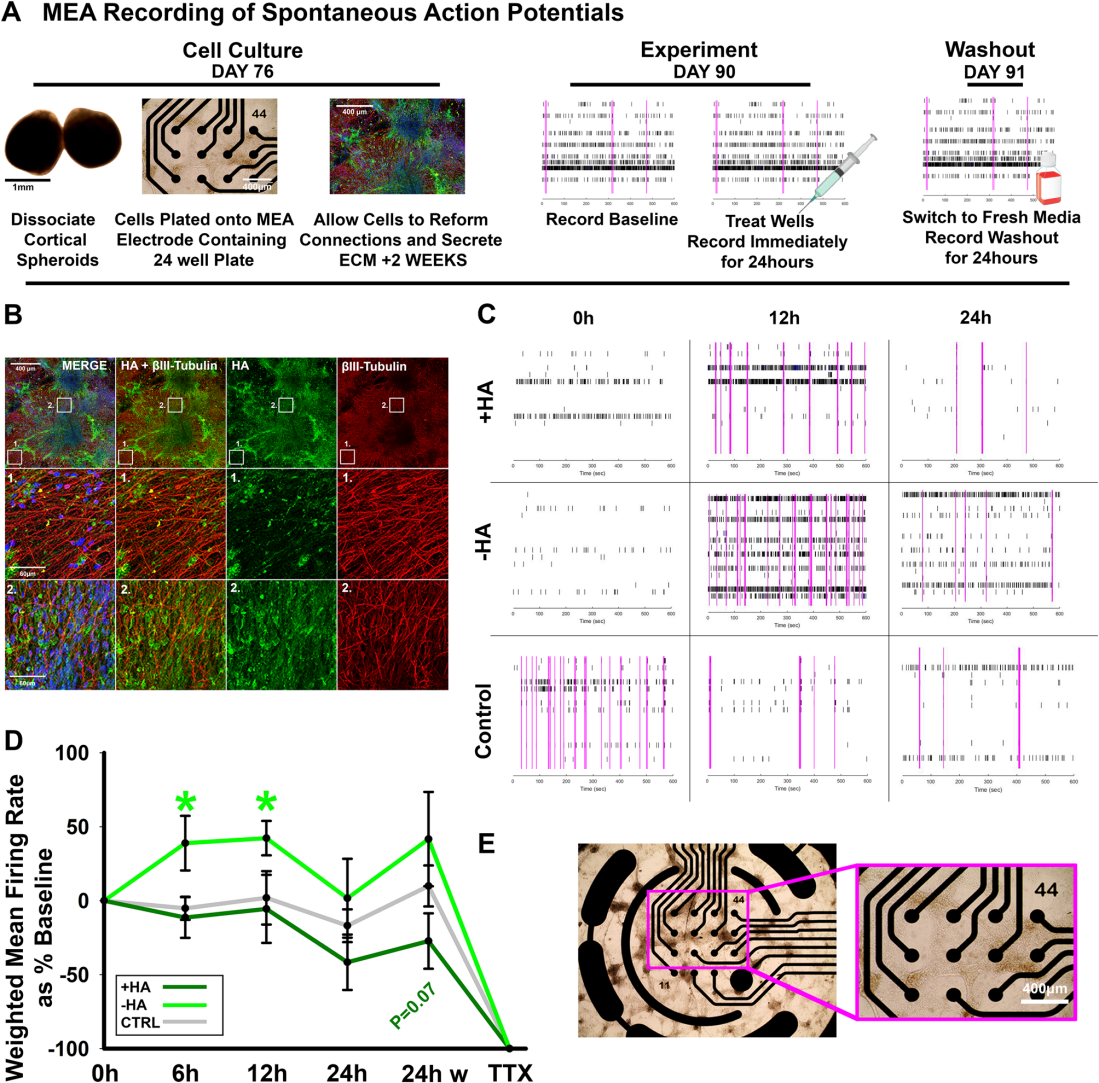
HA regulates spontaneous action potential formation. (**A**) Workflow for MEA experiments. Day 76 spheroids are dissociated enzymatically and plated onto 24-well microelectrode array plates. After 2 weeks, neurons have re-established neural networks and the HA-based ECM. At day 90, the cells are treated and spontaneous action potentials are recorded for 10 min/hour for 24 h. After 24 h, fresh media is added to wells for a washout recording of 10 min/hour for 24 h. (**B**) Representative confocal image for βIII-tubulin (neurons, red), DAPI (nuclei, blue), and HABP (HA, green) for cortical spheroids dissociated at day 76 and imaged at day 90. Middle and bottom panels highlight detail of HABP and βIII-tubulin area. Scale bar top panel: 400 μm, middle and bottom panel: 60 μm. (**C**) Raster plots of spontaneous action potentials in + HA (top), − HA (middle), and control wells (bottom) at 0 h, 12 h, and 24 h after treatment. Each black bar is a single spike, meaning one electrode has fired, pink bars highlight network bursts, where more than one electrode detect simultaneous activity. High network bursting is characteristic of hyperexcitable networks. (**D**) Quantification of the weighted mean firing rate (WMFR) for each treatment at 0 h, 6 h, 12 h, 24 h post treatment, 24 h post washout, and immediately after TTX treatment. Hyaluronidase treatment significantly increased WMFR at 6 h and 12 h following treatment. (**E**) Brightfield image of dissociated spheroids plated for 2 weeks on a microelectrode array. Inset shows association of neurons with electrodes. Scale bar 400 µm.

Our results thus far demonstrate that hyaluronan removal promotes excitatory synapse formation and decreases inhibitory synapse formation (Figs. 4, 5). In an opposite fashion, hyaluronan addition decreases excitatory synapse formation and increases inhibitory synapse formation (Figs. 4, 5). Given the observed changes in synapse formation, we hypothesize that HA functions to attenuate neuronal excitability. Neuronal hyperexitability is cytotoxic and contributes to brain disorders such as autism, epilepsy, and intellectual disability^12,27^. In order to assess the effects of hyaluronan manipulation on neuronal activity, we dissociated 76-day-old cortical spheroids onto microelectrode arrays (Fig. 6A,E). After two weeks on microelectrode surfaces, the dissociated cortical spheroids re-establish neuronal connections and their extracellular matrix (Fig. 6B). After this two-week recovery period, we recorded the baseline neural activity, followed by HA manipulation and recording of neural activity for the 24 h (Fig. 6C), the time period in which we observed changes in synapse formation (Figs. 4, 5). Finally, we used tetrodotoxin treatment to suppress action potential formation, verifying that we were recording spontaneous neural activity (Fig. 6D). After 6–12 h of hyaluronidase treatment, HA removal significantly elevates neural activity over untreated and hyaluronan addition. Conversely the addition of HA has an opposing trend towards depressed neural activity, beginning around 24 h (Fig. 6D). Since cortical spheroids express endogenous HA (Figs. 1, 6B, and Fig. S1), we suggest that the addition of HA requires further time to incorporate into the synaptic ECM and destabilize synaptic structures. Thus, we only begin to observe the effects of HA addition at later timepoints. Overall, these results suggest that hyaluronan prevents the emergence of a hyperexcitable state in developing neural networks. Through these protocols, we have successfully established methodology to acutely manipulate HA levels in cortical spheroids and observe the resulting impact on synapse formation (Figs. 3, 4, 5) and function (Fig. 6).

## Discussion

Through this research, we demonstrate that HA critically regulates synapse formation and function in early human cortical brain development. We established that human cortical spheroids serve as an ideal model for studying the role of the ECM in neural circuit formation. We confirmed that cortical spheroids are capable of producing their own endogenous ECM and explored this in further detail to determine how HA interacts with developing synapses. We found that HA is preferentially present at the synaptic cleft of excitatory synapses compared to inhibitory synapses (Fig. 2 and S4). These data are in contrast to studies conducted during later brain development, when synapses have matured. At these mature synapses, previous data suggests that HA is not present in the synaptic cleft in adult synapses, but instead surrounds and encapsulates synapses^9,49^. This suggests a temporally important regulatory role for HA at developing synapses. It has been shown that HA is highly increased in the brain during this pre-natal developmental window^55^. Furthermore, several other neurodevelopmental processes leading up to synapse formation, such as neurulation and neural crest cell migration, are dependent upon HA concentration^1,3^. Based on the crucial roles of HA leading up to synaptogenesis and our data highlighting its presence at the developing synapse, we hypothesized that HA critically regulates the formation and function of synapses in developing neural networks.

Using human brain spheroids, we were able to address how hyaluronan manipulations directly impact the initial formation of synaptic contacts. Using Nanostring transcriptional analysis, we initially observed increased expression of synaptic mRNAs in response to hyaluronan removal. These synaptic transcripts include *SNAP91, SLC4A10, SHANK2,* and *GRIA2*. *SNAP91* (also referred to as F1-20) expression has been associated with excitatory synapse maturation^56^, suggesting that hyaluronidase may accelerate excitatory synapse development. Similarly, overexpression of *GRIA2*, a glutamate receptor subunit is sufficient to induce dendritic spine formation^57^. Furthermore, *SLC4A10* promotes neuronal excitability, with *slc4a10-/-* mice exhibiting reduced epileptic seizure susceptibility^58^, while *SHANK2* regulates neuronal excitability through NMDA receptor function^59^. Together, the affected transcripts suggest that hyaluronan removal promotes a hyperexcitable state, which we demonstrated through further analysis of synapse formation and function. HA manipulation alone was sufficient to alter the excitatory to inhibitory synaptic ratio and the resulting synaptic activity (Figs. 3, 4, 5 and 6).

Our results establish a new role for HA during the formation of synapses, that further highlights HA as a critical regulator of synaptic plasticity throughout brain development. For example, HA has previously been described as a regulator of neuroplasticity through perineuronal nets. Depletion of HA through degradation of perineuronal nets in mice revealed increased amplitude of action potentials at the excitatory postsynaptic terminal^60^. However, perineuronal nets form during postnatal week 3–5 in mice^61^, a timepoint corresponding to about 4–11 years in humans^62^. Perineuronal nets stabilize mature synapses on a subset of inhibitory neurons by restricting neurite growth and synapse formation^7,8^. The dense ECM meshwork of perineuronal nets is thought to protect the specified neuron from hyperexcitability. However, HA is also present in the interstitial space between neurons^12^. In our data, we observed HA concentrated in the developing cortical plate of brain spheroids (Fig. 1), where a significant portion localized to nascent excitatory synapses (Fig. 2) and regulated the emerging neural activity (Fig. 6). Consistent with our data that HA can regulate brain physiology independent of the formation of perineuronal nets, HAS3^−/−^ mice exhibited unperturbed perineuronal nets, yet these mice suffered hippocampal seizures^12^. Together with our results, these data further demonstrate that HA can regulate neuronal excitability independent of peri-neuronal nets, by directly affecting synaptic signaling events^16^.

To appreciate the diverse mechanisms by which HA may regulate synapse formation and function, it is necessary to evaluate the physiological and pathophysiological roles of the HA-based ECM in other tissue systems. HA has been most heavily explored in cartilage, where it provides strength, maintains space, and resists shear forces and compression. HA is required for these mechanical functions, which rely on the retention of bound proteoglycans^63^. In cartilage, HA has additionally been shown to regulate chondrocyte maturation^18,64^. In skin keratinocytes, HA-CD44 interactions have been shown to affect proliferation, survival, migration, cell–cell adhesion, and differentiation^65^. Similarly, in metastatic breast cancer, the interaction of HA with the CD44 receptor mediates tumor cell migration^1,2,4^. Translating these diverse roles into brain physiology, it is known that HA is required for cell migration during neurulation and that HA has effects on proliferation and differentiation in the hippocampal sub-granular zone of mice^12,16^. Furthermore, HA deficiency in mouse HAS isoform knockouts prevents extracellular water retention and reduces the size of the extracellular space, similar to the space-filling role of HA in cartilage; this reduced extracellular space elicits epileptic seizures through a postulated increase in neurotransmitter diffusion^12,16^. Notably, restored osmolarity was sufficient to reduce neuronal excitability^12^. Consistent with the space-filling role of HA, our data supports a model whereby HA restricts the available space for pre- and post-synaptic membranes to form and contact one another. In this model where HA serves a space-filling role, one would predict that HA would predominantly suppress the formation of excitatory synapses, which form on post-synaptic actin-rich protrusions, as opposed to inhibitory synapses which form along the dendrite. Multiple lines of evidence suggest that HA preferentially restricts excitatory synapse formation, including the selective enrichment of HA at excitatory synapses (Fig. 2), and HA-mediated reductions in excitatory synapse area (Fig. 4) as contrasted with increased inhibitory synapse formation with increasing HA levels (Fig. 5). Since HA does not restrict, but rather promotes, inhibitory synapse formation, we suggest that these effects may be indirectly driven by competition for shared synaptic proteins. For example, competition for the adhesive neurexin-neuroligin complex regulates the balance between excitatory to inhibitory synapse formation and signaling^17^. These inverse changes in synapse formation exacerbate imbalances in the emerging ratio between excitatory and inhibitory signaling, resulting in a hyperexcitable state in response to HA removal (Fig. 6).

While most research on the loss of HA in pathological conditions focuses on the loss of physical extracellular space, HA also regulates intercellular signaling through its interaction with its known cell surface receptors, RHAMM and CD44, of which the most abundant is CD44^8^. Both RHAMM and CD44 are expressed by neurons^3^. While RHAMM is expressed in neurons, it predominantly regulates cell migration and cell cycle in astrocytes and microglia, where it has been shown to interact with microtubules and the actin cytoskeleton^66^. The predominant HA receptor, CD44, is found in neurons, astrocytes and neural progenitor cells, and has been visualized in our model (Fig. 1). The signaling mechanism of this transmembrane receptor is not yet fully understood. HA binds to N-terminal motifs of CD44 that act as loading sites, giving rise to clusters as it binds to HA outside the cell^67^. This alone is hypothesized to cause cellular response through changes in force on the cell surface that are balanced by intercellular attachment of CD44 to the actin cytoskeleton through ERM complexes^18,20,65^. CD44 overexpression has been linked to changes in astrocyte morphology. CD44 has also been linked to RhoGTPase actin cytoskeletal regulators, Rac1 and RhoA, in astrocytes as well as other systems such as breast cancer cells and keratinocytes^21,68^. However, a canonical signaling pathway for CD44 has not yet been agreed upon, and CD44 may also signal outside of HA binding. For example, MMP-mediated cleavage of the extracellular domain of CD44 can regulate CD44 signaling pathways^68^. Thus, while many studies have examined the role of ECM receptors, such as CD44, fewer have actually examined the direct impact of ECM alterations on synapses as we have done.

Although further work is needed to parse out the mechanism by which HA orchestrates neural circuit formation, we have demonstrated significant synaptic changes at the mRNA, protein, and functional levels in response to HA manipulations. Through this research, we demonstrated a novel role for the HA-ECM in the establishment of the E/I ratio of developing networks. In particular, HA removal was sufficient to drive a hyperexcitable state, which is characteristic of neurodevelopmental disorders, including epilepsy, intellectual disability, and autism spectrum disorders^12,35,40^. Conversely, our observation that HA decreases excitatory synapse formation could have implications for aging and Alzheimer’s Disease, both of which exhibit HA accumulation and synapse loss^69–72^. In particular, aging is associated with loss of synapses that form on highly plastic thin spines^69^, which resemble the filopodia-like spine precursors involved in prenatal synaptogenesis^30,48,73^. Thus, the findings in the study could have relevance for a spectrum of neurologic conditions, from neurodevelopmental disorders to age-related cognitive decline. In conclusion, our data is the first to demonstrate direct integration of HA between the pre- and post-compartments of developing excitatory synapses. Further modulation of HA and associated ECM components will advance our understanding of the physiological roles of ECM in early brain development and identify potential therapeutic targets for neurological disorders.

## Supplementary information

Supplementary figure

Supplementary figure

Supplementary figure

Supplementary figure

Supplementary figure legends

Supplementary tables
