# Supplementary figures and images for: Hyaluronan regulates synapse formation and function in developing neural networks
Source: Sci Rep. 2020 Oct 5;10:16459. doi: 10.1038/s41598-020-73177-y (PMC7536407; doi:10.1038/s41598-020-73177-y)

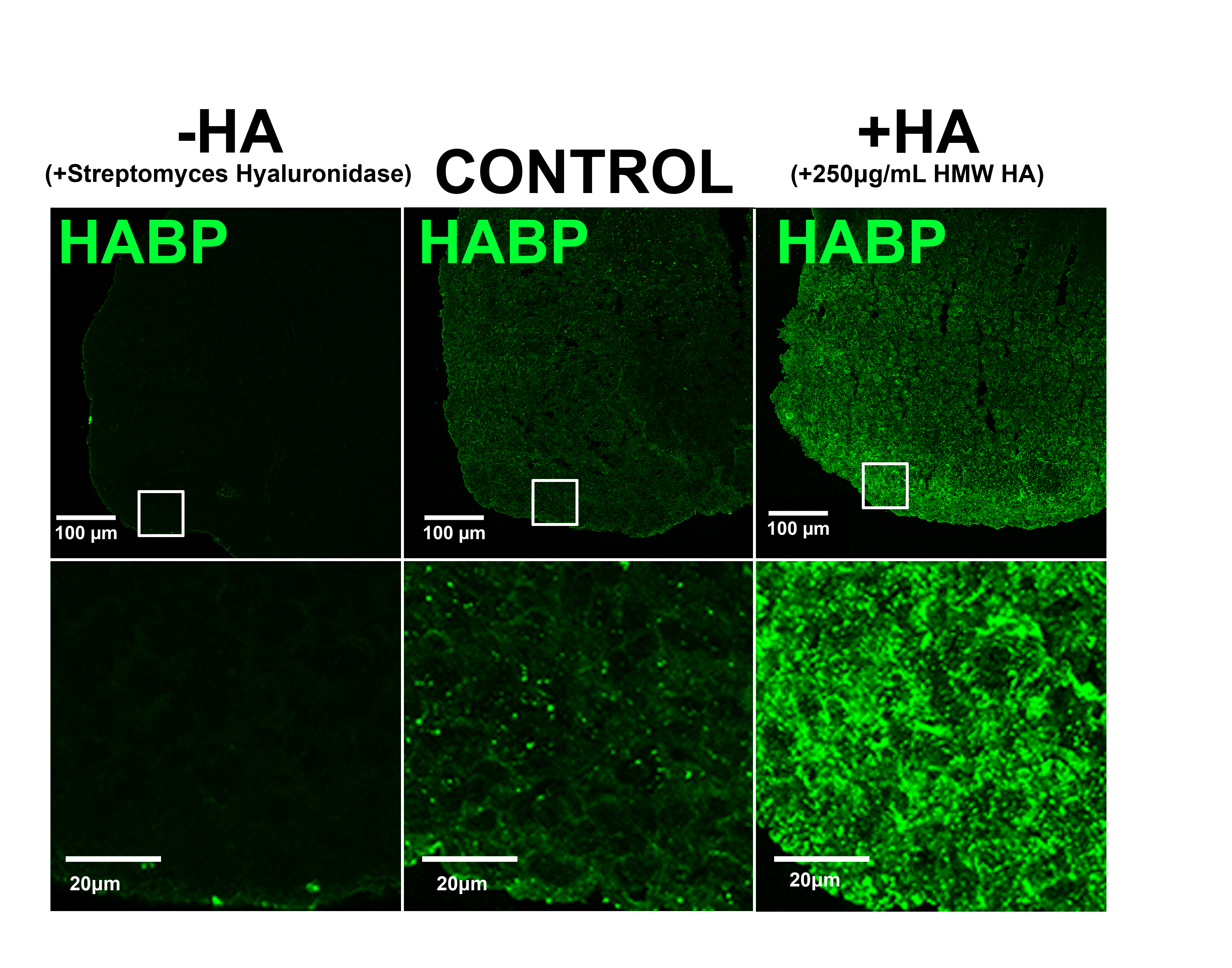

Supplement: Supplementary file 1 — Supplementary figure [file 41598_2020_73177_MOESM1_ESM.tif]

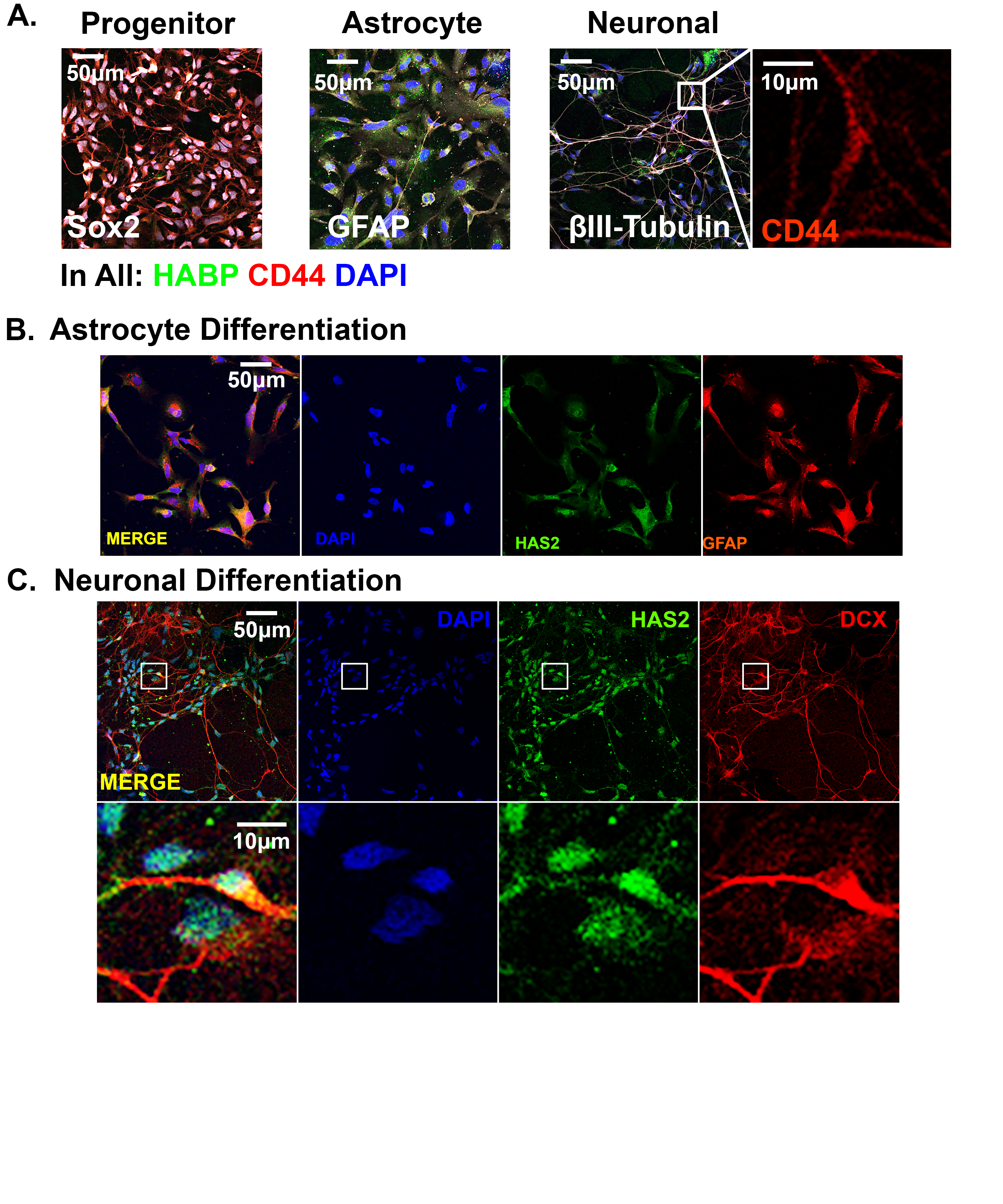

Supplement: Supplementary file 2 — Supplementary figure [file 41598_2020_73177_MOESM2_ESM.tif]

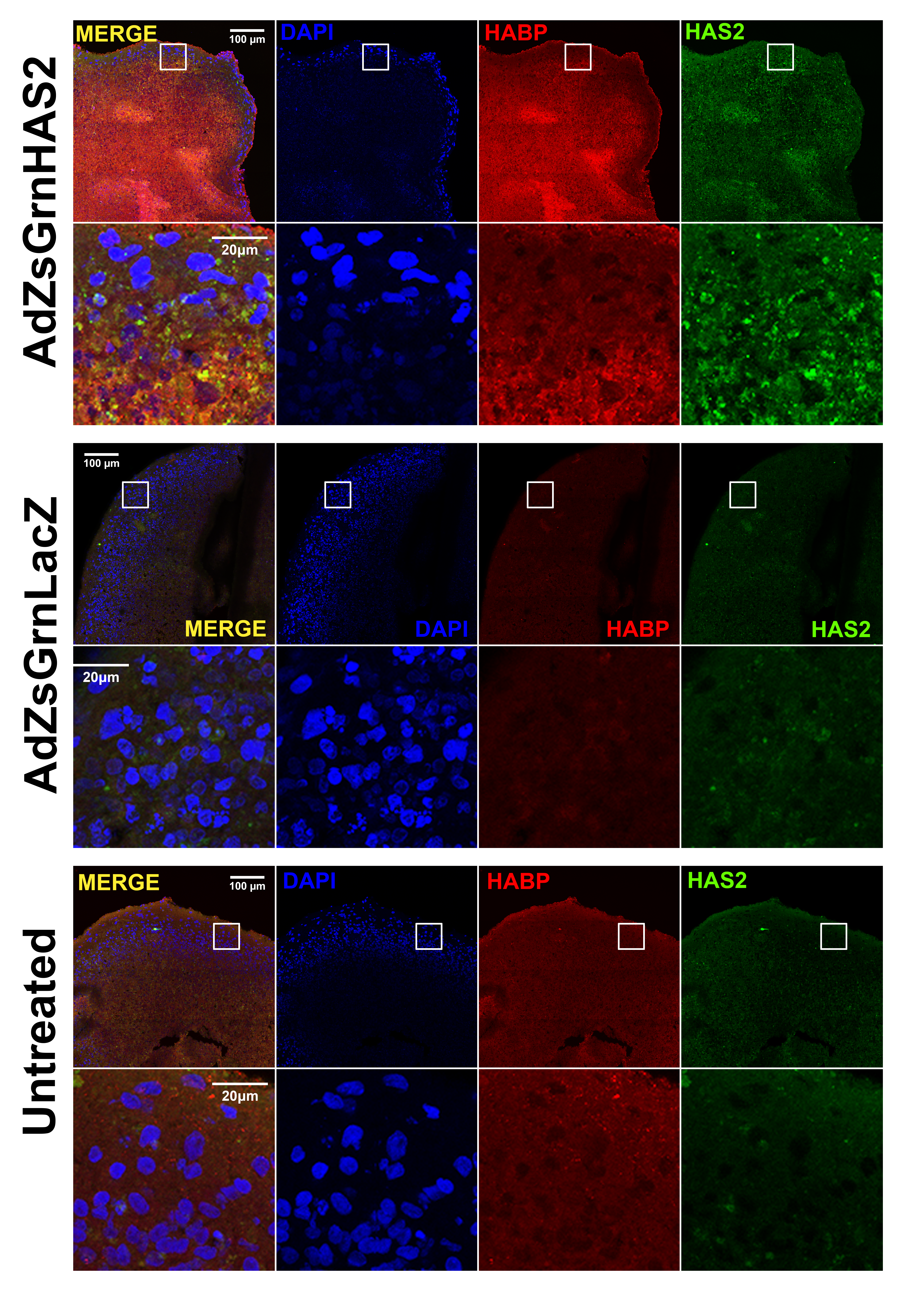

Supplement: Supplementary file 3 — Supplementary figure [file 41598_2020_73177_MOESM3_ESM.tif]

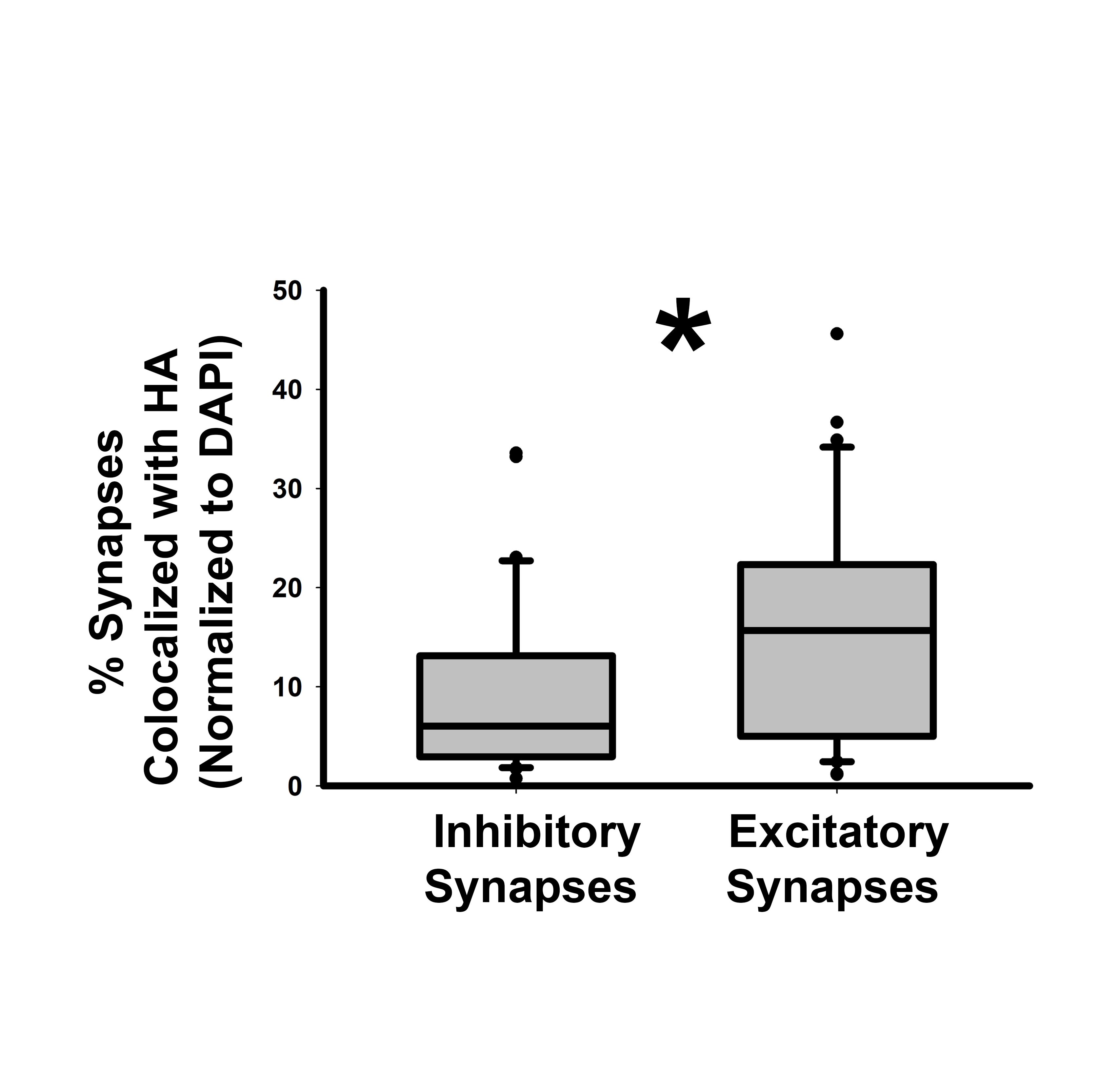

Supplement: Supplementary file 4 — Supplementary figure [file 41598_2020_73177_MOESM4_ESM.tif]
