# Supplementary material for: Hyaluronan regulates synapse formation and function in developing neural networks
Source: Sci Rep. 2020 Oct 5;10:16459. doi: 10.1038/s41598-020-73177-y (PMC7536407; doi:10.1038/s41598-020-73177-y)
Supplement: Supplementary file 5 — Supplementary figure legends [file 41598_2020_73177_MOESM5_ESM.docx]

Hyaluronan Regulates Synapse Formation and Function in Developing Neural Networks

Emily Wilson^1^, Warren Knudson^1^, Karen Newell-Litwa^1*^

^1^Department of Anatomy and Cell Biology, Brody School of Medicine, East Carolina University, Greenville, NC, USA

*Correspondence to [litwak16@ecu.edu](mailto:litwak16@ecu.edu)

**Supplemental Figure Legends:**

**Supplemental Figure 1**. **Manipulation of HA Levels**. **Top Panel.** 10μm-thick cryosections of 90-day-old cortical spheroids were stained for HA (HABP) after 24 hours of treatment with purified HA (+HA) or streptomyces hyaluronidase (-HA). **Bottom Panel.** Section of the cortical plate at increased magnification. Scale bar of top panel: 100μm, bottom panel: 20μm.

**Supplemental Figure 2. 2D Differentiated Cultures Express HA-ECM Machinery. A.** NPCs (left) differentiated into neurons (middle) and astrocytes (right) express cell-type specific markers (white), HA (green) and CD44 (red), nuclei in blue (DAPI). Scale bar: 50μm. Middle inset highlights CD44 expression in neurons. Scale bar: 10μm. **B**. Differentiated astrocytes express GFAP (red), HAS2 (green), and nuclei (DAPI, blue). Scale bar: 50μm. **C.** Differentiated neurons express doublecortin (DCX, red) and HAS2 (green), nuclei in blue (DAPI). Scale bar: 50μm. Bottom panel highlights a neuron cell body and proximal neuronal processes. Scale bar: 10μm.

**Supplemental Figure 3**. **Manipulation of HAS2 Levels. Top.** 48hour viral transduction of day 90 cortical spheroids with HAS2 overexpression plasmid AdZsGrnHAS2, stained for DAPI (blue) HABP (red) and HAS2 (green). **Middle**. 48hour viral transduction of day 90 cortical spheroids with control LacZ plasmid AdZsGrnLacZ, stained for DAPI (blue) HABP (red) and HAS2 (green). **Bottom**. Untreated day 90 cortical spheroids stained for DAPI (blue) HABP (red) and HAS2 (green). Note that compared to LacZ and untreated controls, HAS2 transduction increases HAS2 expression as well as corresponding HA levels.

**Supplemental Figure 4. Synapse Association with HA.** To account for the disproportionate number of excitatory synapses in our cortical spheroids (Fig. 2), we also quantified the percentage of inhibitory and excitatory synapses that associated with HA. The percentage of excitatory synapses that exhibit HA association is significantly greater than the percentage of inhibitory synapses that associate with HA.
