# Supplementary material for: Hyaluronan regulates synapse formation and function in developing neural networks
Source: Sci Rep. 2020 Oct 5;10:16459. doi: 10.1038/s41598-020-73177-y (PMC7536407; doi:10.1038/s41598-020-73177-y)
Supplement: Supplementary file 6 — Supplementary tables [file 41598_2020_73177_MOESM6_ESM.docx]

**Supplemental Tables**

**S. Table 1. Primary Antibodies**

| **Primary Antibody** | **Brand** | **Catalog Number** | **Host** | **Class** | **Dilution** | **Concentration Used** |
| --- | --- | --- | --- | --- | --- | --- |
| ß-III Tubulin | R&D Systems | MAB1195  RRID:AB_10673030 | Mouse IgG | Monoclonal | 1:500* | 1μg/mL |
| Doublecortin | abcam | ab153668  RRID:AB_2728759 | Chicken IgY | Polyclonal | 1:100* | 2μg/mL |
| Gephyrin | Synaptic Systems | 147 RRID:AB_887716 | Mouse  IgG | Monoclonal | 1:500* | 1μg/mL |
| Gephyrin | Abcam | Ab32206  RRID:AB_2112628 | Rabbit IgG | Polyclonal | 1:500* | 1μg/mL |
| GFAP | Synaptic Systems | 173 002  RRID:AB_887720 | Rabbit  IgG | Polyclonal | 1:500* | 1μg/mL |
| GFAP | Calbiochem | NE1015 | Mouse  IgG | Monoclonal | 1:1000* | 1μg/mL |
| HABP | Calbiochem | 385911 | Bovine | Biotinylated | -- | 4μg/mL |
| HAS2 | ThermoFisher Scientific | MA5-17087  RRID:AB_2538558 | Mouse  IgG | Monoclonal | 1:200* | 1μg/mL |
| Hermes-1 | DSHB | -- | Rat  IgG | Monoclonal | 1:50* | 1μg/mL |
| PSD-95 | Santa Cruz | sc-32291  RRID:AB_628113 | Mouse  IgG | Monoclonal | 1:50* | N/A |
| Sox2 | Abcam | ab79351  RRID:AB_10710406 | Mouse  IgG | Monoclonal | 1:500* | 1μg/mL |
| VGAT | Synaptic Systems | 131 004  RRID:AB_887873 | Guinea Pig IgG | Polyclonal | 1:1000* | N/A |
| Vglut-1 | Synaptic Systems | 135 304  RRID:AB_2621384 | Guinea Pig IgG | Polyclonal | 1:1000* | N/A |

***IHC**

**S. Table 2 Secondary Antibodies**

***IHC**

| **Secondary Antibody** | **Brand** | **Catalog Number** | **Host** | **Class** | **Dilution** | **Concentration Used** |
| --- | --- | --- | --- | --- | --- | --- |
| Anti-Chicken Alexa Fluor 488 | Invitrogen | A-11039  RRID:AB_142924 | Goat IgG | Polyclonal | 1:500* | 4μg/mL |
| Anti-Guinea Pig Alexa Fluor 568 | Invitrogen | A-11075  RRID:AB_141954 | Goat IgG | Polyclonal | 1:500* | 4μg/mL |
| Anti-Guinea Pig Alexa Fluor 647 | Invitrogen | A-21450  RRID:AB_141882 | Goat IgG | Polyclonal | 1:500* | 4μg/mL |
| Anti-Mouse Alexa Fluor 488 | Invitrogen | A-11001  RRID:AB_2534069 | Goat IgG | Polyclonal | 1:500* | 4μg/mL |
| Anti-Mouse Alexa Fluor 568 | Invitrogen | A-11004  RRID:AB_2534072 | Goat IgG | Polyclonal | 1:500* | 4μg/mL |
| Anti-Mouse Alexa Fluor 647 | Invitrogen | A-21235  RRID:AB_2535804 | Goat IgG | Polyclonal | 1:500* | 4μg/mL |
| Anti-Mouse Alexa Fluor 488 IgM | Invitrogen | A-21042  RRID:AB_141357 | Goat IgG | Polyclonal | 1:500* | 4μg/mL |
| Anti-Rabbit Alexa Fluor 488 | Invitrogen | A-11008  RRID:AB_143165 | Goat IgG | Polyclonal | 1:500* | 4μg/mL |
| Anti-Rabbit Alexa Fluor 568 | Invitrogen | A-11011  RRID:AB_143157 | Goat IgG | Polyclonal | 1:500* | 4μg/mL |
| Anti-Rabbit Alexa Fluor 647 | Invitrogen | A-21245  RRID:AB_2535813 | Goat IgG | Polyclonal | 1:500* | 4μg/mL |
| Streptavidin 568 | Molecular Probes | S11226 | N/A | N/A | 1:1000 | 2μg/mL |
| Streptavidin  488 | Molecular Probes | S11223 | N/A | N/A | 1:1000 | 2μg/mL |
| Atto 488 | Sigma Aldrich | 18772  RRID:AB_1137637 | Rabbit IgG | Polyclonal | 1:500 | 4μg/mL |

**S. Table 3. Figure 2 HA colocalized with Synapses p Values.** ** p < 0.001 * p < 0.05

| **Figure** | **Comparison** | **Statistical Analysis** | **P Value** |
| --- | --- | --- | --- |
| **2C** | Excitatory vs Inhibitory Synaptic Marker Colocalized with HABP | Two-Tailed T-Test | P = 0.027 |
| **2D** | HABP colocalized with Excitatory vs Inhibitory Synaptic Marker | Two-Tailed T-Test | P = <0.001 |
| **2F** | Distance of HABP to VGLUT vs Distance of HABP to PSD95 | Two-Tailed T-Test | P=0.070 |

**S. Table 4. Figure 3 Nanostring p Values.** ** p < 0.001 * p < 0.0

| **Figure** | **Pathway Ratio Vs Average Ratio** | **Statistical Analysis** | **P value** |
| --- | --- | --- | --- |
| 3B | Chromatin Modification | Two-Tailed T-Test | P = 0.009 |
|  | Neuronal Cytoskeleton | Two-Tailed T-Test | P = 0.445 |
|  | Trophic Factors | Two-Tailed T-Test | P = 0.093 |
|  | **Pathway Ratio Vs If No Difference Between Groups (ratio=1)** |  |  |
| 3B | Chromatin Modification | Two-Tailed T-Test | P=0.113 |
|  | Neuronal Cytoskeleton | Two-Tailed T-Test | P=0.406 |
|  | Trophic Factors | Two-Tailed T-Test | P=0.396 |
| 3C | **-HA vs +HA** | Two-Tailed T-Test | **P value** |
|  | GRIA 2 | Two-Tailed T-Test | P=0.00499 |
|  | SHANK2 | Two-Tailed T-Test | P=0.00156 |
|  | SLC4A10 | Two-Tailed T-Test | P=0.00842 |
|  | SNAP91 | Two-Tailed T-Test | P=0.00295 |

**S. Table 5. Figure 4 Excitatory Synapse p Values .** ** p < 0.001 * p < 0.05

| **Figure** | **Comparison** | **Statistical Analysis** | **P value** |
| --- | --- | --- | --- |
| 4B | **_CTRL HABP vs +HA HABP_** | Two-Tailed T-Test | P = <0.001 |
| 4B | **_CTRL HABP vs -HA HABP_** | Two-Tailed T-Test | P = <0.001 |
| 4B | **_CTRL PSD95 vs +HA PSD95_** | Two-Tailed T-Test | P = 0.020 |
| 4B | **_CTRL PSD95 vs -HA PSD95_** | Two-Tailed T-Test | P = <0.001 |
| 4B | **_CTRL VGLUT1 vs +HA VGLUT1_** | Two-Tailed T-Test | P = 0.075 |
| 4B | **_CTRL VGLUT1 vs -HA VGLUT1_** | Two-Tailed T-Test | P = 0.003 |
| 4C | **_CTRL E SYN vs +HA E SYN_** | Two-Tailed T-Test | P = 0.008 |
| 4C | **_CTRL SYN vs -HA E SYN_** | Two-Tailed T-Test | P = <0.001 |

**S. Table 6. Figure 5 Inhibitory Synapse p Values.** ** p < 0.001 * p < 0.05

| **Marker** | **Comparison** | **Statistical Analysis** | **P Value** |
| --- | --- | --- | --- |
| Gephyrin | 0-9%vs 10-19% | Two-Tailed T-Test | P=0.047 |
|  | 0-9%vs 20-29% |  | P = 0.001 |
|  | 0-9% vs 30-39% |  | P=0.131 |
|  | 0-9% vs 40-49% |  | P = <0.001 |
|  | 0-9% vs 50-59% |  | P = <0.001 |
|  | 0-9% vs 60-69% |  | P = <0.001 |
|  | 0-9% vs 70-79% |  | P = <0.001 |
|  | 0-9% vs 80-89% |  | P = <0.001 |
|  | 0-9% vs 90-99% |  | P = <0.001 |
|  | 0-9% vs 100+% |  | P = <0.001 |
| VGAT | 0-9%vs 10-19% | Two-Tailed T-Test | P=0.936 |
|  | 0-9%vs 20-29% |  | P=0.979 |
|  | 0-9% vs 30-39% |  | P=0.015 |
|  | 0-9% vs 40-49% |  | P=0.306 |
|  | 0-9% vs 50-59% |  | P=0.035 |
|  | 0-9% vs 60-69% |  | P=0.792 |
|  | 0-9% vs 70-79% |  | P=0.937 |
|  | 0-9% vs 80-89% |  | P=0.436 |
|  | 0-9% vs 90-99% |  | P=0.116 |
|  | 0-9% vs 100+% |  | P = <0.001 |
| Colocalized Synaptic Marker | 0-9%vs 10-19% | Two-Tailed T-Test | P=0.055 |
|  | 0-9%vs 20-29% |  | P=0.006 |
|  | 0-9% vs 30-39% |  | P=0.576 |
|  | 0-9% vs 40-49% |  | P=0.001 |
|  | 0-9% vs 50-59% |  | P = <0.001 |
|  | 0-9% vs 60-69% |  | P = <0.001 |
|  | 0-9% vs 70-79% |  | P=0.002 |
|  | 0-9% vs 80-89% |  | P = <0.001 |
|  | 0-9% vs 90-99% |  | P = <0.001 |
|  | 0-9% vs 100+% |  | P = <0.001 |

**S. Table 7. Figure 6D Multi-Electrode Array Analysis p Values.**

** p < 0.001 * p < 0.05

| **Statistical Analysis** | **Comparison** | **P value** |
| --- | --- | --- |
| ANOVA | -HA vs +HA | P=0.005 |
|  | -HA vs Control | P=0.052 |
|  | Control vs +HA | 0.398 |
| Two-Tailed T-Test | 6hr Ctrl Vs 6hr -HA | 0.0359 |
|  | 12hr Ctrl vs -HA | 0.0421 |
|  | 12hr Ctrl vs -HA | 0.283 |
